# Sharing data from the Ecosystem Services for Poverty Alleviation programme

**DOI:** 10.1038/sdata.2018.137

**Published:** 2018-07-03

**Authors:** K. Homewood, K. Schreckenberg

**Affiliations:** 1 Anthropology, University College London, Gower Street, London WC1E 6BT, UK; 2 Geography, King’s College London, Bush House, 30 Aldwych, London WC2B 4BG, UK

**Keywords:** Sustainability, Developing world

The 2005 Millennium Ecosystem Assessment^1^ suggested that while there have been substantial gains in human wellbeing in recent decades, these have been achieved at the expense of high, often irreversible levels of ecosystem degradation. The linkages between ecosystem services and poverty alleviation, however, are complex and poorly understood. In many cases, gains in well-being have gone hand-in-hand with rising inequalities and increasing vulnerability of the more marginalised to environmental shocks and stresses. The Ecosystem Services for Poverty Alleviation (ESPA, https://www.espa.ac.uk/) interdisciplinary research programme aimed to work towards the Sustainable Development Goals^2^ by giving decision-makers and natural resource users the evidence they need to address the challenges of combining sustainable ecosystem management with poverty reduction. ESPA set out to:

- build a strong evidence base on how human use interacts with ecosystem services, and their dynamics and management;
- develop innovative, interdisciplinary research and methodologies, so decision-makers may predict socio-ecological responses to complex social and economic trends;
- get research into practice, by engaging with policymakers, practitioners and decision-makers;
- use international research partnerships to build research capacity around the global South.

The scope of ESPA’s research into different dimensions of ecosystem services, and the social and ecological implications of their management, are summarised in Fig. 1.

This collection highlights a series of ESPA-funded projects contributing original, interdisciplinary datasets and analytical approaches on social-ecological systems in Asia, Africa and Latin America. They cover ecosystems ranging from montane headwater catchments, through arid and semi-arid rangelands, mesic farmlands and wetlands, to coastal deltas, mangrove and island forests. This collection makes available to a wider scientific audience a set of rigorously compiled and carefully curated interdisciplinary datasets characterising interactions in these social-ecological systems. Each dataset offers considerable potential for further analysis both in its own right and in conjunction with new data, not least as baselines for future studies of the impacts of environmental change and interventions. The data descriptor papers presented here fall into two categories. The first focuses on drivers and outcomes of variability and change in the biophysical dimensions of key social-ecological systems, while the second documents the social implications of these changes.

Decision-making and resource-use planning need data on changes in environment, resource availability and use, and on the drivers of those changes. The first paper by Ochoa-Tocachi *et al*.^3^ addresses measurement of the biophysical features underpinning social-ecological systems, in order to inform land use planning and evaluate the impacts of environmental interventions. This paper presents hydrological measurements from multiple paired catchments, encompassing a wide range of ecosystem types and management systems across three major biomes of the Andean montane region (*páramo*, *jalca*, and *puna* as well as montane forest) and three countries (Bolivia, Peru, Ecuador). By comparing intervention and control sites, the study was able to attribute observed effects to particular land use changes, restoration or water harvesting interventions. Datasets document rainfall, streamflow, and catchment shape, drainage, elevation, topography, soils, land cover and land use, including derived indices of change, under different management approaches, in water yield, water balance, streamflow magnitude, timing and flashiness; and ratio of baseflow to total flow. These data can be used to analyse the impact of human activities on watersheds, and to guide decision-making and governance aimed at reducing poverty.

Beyond documenting the spatial and biophysical underpinnings of social-ecological systems, ESPA set out to establish an evidence base on how different groups of people (by wealth, or occupation, or other dimensions of social difference) are impacted by and respond to changes in their environment. The second, larger group of papers in this collection deals primarily with datasets reporting the lives, livelihoods and wellbeing of local communities in the face of environmental change or intervention. Stakeholder participation is an essential ingredient of this set of papers. All involve in-depth, two-way participation, with local users shaping the ways research questions are formulated and research undertaken, as well as volunteering the information recorded, and validating those data through their feedback. All employ ethics scrutiny of processes for working with local respondents^4^, and free prior informed consent procedures adapted to prevailing literacy and local sensitivities.

The different studies illustrate a range of approaches in terms of how to conceptualise relationships between local users and ecosystem services, in terms of research design, and in terms of practical purpose. Adams *et al*.^5^ use an initial spatial land cover classification, comprising seven dominant production systems in the Ganges-Meghna-Brahmaputra delta: rainfed or irrigated farming, freshwater prawn or saltwater shrimp farming, and riverine, mangrove or offshore systems. Their household survey datasets then form the basis for analysis of multidimensional wellbeing associated with particular farming, fishing or other livelihoods and social-ecological systems.

In the other papers, household survey datasets are used to evaluate implications of specific ecosystem services interventions, including conservation agriculture and wildlife management areas, and are structured accordingly. In their study of subsidy payments in Malawi, Bell *et al*.^6^ apply a classic formal agricultural research experimental design to allow causal attribution. An initially purposive sample design to choose intervention and matching control sites is followed by random sampling stratified by location and involvement in the subsidy programme, drawing on formal lists of farmers. Where formal lists are not available, projects have to undertake baseline surveys to establish sample frames. These baseline datasets are themselves a rich source of possible investigation. For example, the data collected by Bluwstein *et al*.^7^ allow for analysis of the change in wealth rank through time for 13578 Tanzanian households, potentially contributing to our understanding of changing prosperity in rural Tanzania. Subsequent in-depth survey sample datasets include a couple of thousand households.

Different studies all follow similar principles of questionnaire, survey and interview design, building on mixed methods and using qualitative approaches to establish context, identify key issues and variables to be evaluated. Local language issues are addressed in different ways according to circumstances, but all rely on extensive discussion, training and role play to ensure data collectors share the same in-depth grasp of underlying principles, general thrust and specific meaning of individual terms used and questions asked. All consider quality control issues, with built in checks at various stages. The different studies capture different but overlapping sets of key variables according to their research question. All record household demographic composition and socioeconomic status (based on wealth ranking^7^ or asset indices); natural resource use practices; livelihoods and income; perceptions of wellbeing and of its change, both through time and with respect to specific interventions; and in one case^5^ biomedical status (body mass index, blood pressure, etc.); perceptions of the interventions and of the benefits (or disadvantages) of involvement. Each of these papers represents a useful baseline dataset against which the implications of future environmental change in general and of current environmental interventions in particular can be measured.

The overarching message of ESPA's research is that policy and programmes that use or regulate environmental resources inevitably carry implications for human wellbeing and often bear hidden costs, which are unevenly distributed within and between groups^8^. These implications and potential human costs must be understood and addressed through open, just and democratic processes^9^. This can only happen on the basis of detailed, unbiased data on the social, physical, financial and other implications of processes such as climate change, and of interventions in agriculture or natural resource management. Most importantly, these data must be disaggregated for different places and groups of people. The ESPA programme supported a range of studies producing such evidence, and this ESPA collection is a step towards making such datasets available for wider use, addressing a range of social-ecological systems, and illustrating a range of ways of going about such research. Additional data descriptors arising from other ESPA-funded projects will be added to this collection in the future. We hope that these studies will be both a valuable baseline and a strong stimulus for further work.

## Additional information

**How to cite this article**: Homewood, K. & Schreckenberg, K. Sharing data from the Ecosystem Services for Poverty Alleviation programme. *Sci. Data* 5:180137 doi: 10.1038/sdata.2018.137 (2018).

**Publisher’s note**: Springer Nature remains neutral with regard to jurisdictional claims in published maps and institutional affiliations.

## Figures and Tables

**Figure 1:**
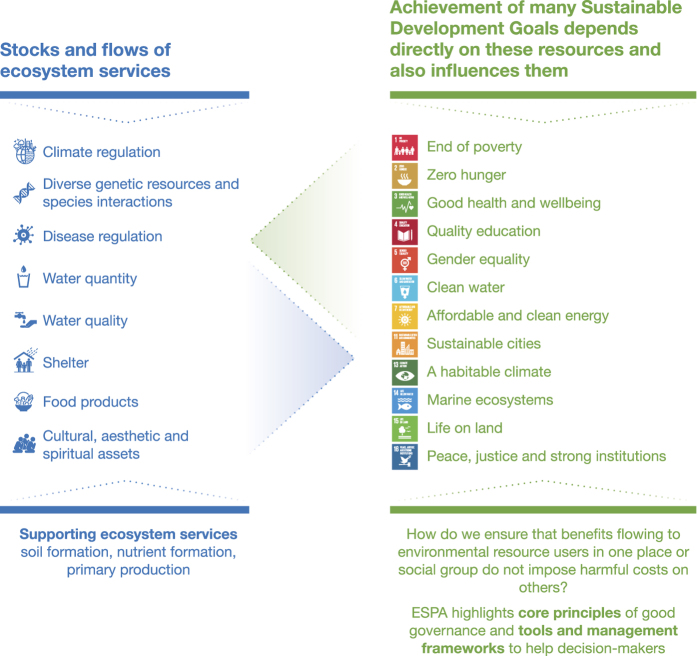
Interactions and trade-offs among outcomes for human wellbeing. Source: ESPA, 2017 (ref. 9).

## References

[b1] Millennium Ecosystem Assessment (Program). Ecosystems and human well-being: synthesis (Island Press, 2005).

[b2] United Nations. Transforming our world: the 2030 Agenda for Sustainable Development. Report No. A/RES/70/1 (2015).

[b3] Ochoa Tocachi B. F. *et al.* High-resolution hydrometeorological data from a network of headwater catchments in the tropical Andes. Sci. Data 5, 180080 (2018). 10.1038/sdata.2018.80 PMC6029571 29969116

[b4] Ethical Guidelines for good research practice. Association of Social Anthropologists of the UK and Commonwealth https://www.theasa.org/downloads/ASA%20ethics%20guidelines%202011.pdf (2011).

[b5] Adams H. *et al.* Spatial and temporal dynamics of multidimensional well-being, livelihoods and ecosystem services in coastal Bangladesh. Sci. Data 3, 160094 (2016). 27824340 10.1038/sdata.2016.94 PMC5100685

[b6] Bell A. R. *et al.* Smart subsidies for catchment conservation in Malawi. Sci. Data 5, 180113 (2018). 10.1038/sdata.2018.113 PMC6029568 29969112

[b7] Bluwstein J. *et al.* A quasi-experimental study of impacts of Tanzania’s Wildlife Management Areas on rural livelihoods and wealth. Sci. Data 5, 180087 (2018). 10.1038/sdata.2018.87 PMC6029570 29969117

[b8] Mace G., Schreckenberg K. & Poudyal M. in Ecosystem Services and Poverty Alleviation (eds Kate Schreckenberg, Georgina Mace, & Mahesh Poudyal) Ch. 19 (Routledge, 2018).

[b9] An environment for wellbeing: Pathways out of poverty, Policy messages from the ESPA programme. The Ecosystem Services for Poverty Alleviation (ESPA) programme http://www.espa.ac.uk/ESPA-policy-summary (2018).

